# Incorporation of Carbocyclic Moieties into Polymer Structure: A Powerful Way to Polymers with Increased Microporosity

**DOI:** 10.3390/polym17081100

**Published:** 2025-04-18

**Authors:** Maxim A. Zotkin, Kirill V. Zaitsev, Dmitry A. Alentiev

**Affiliations:** 1 A.V. Topchiev Institute of Petrochemical Synthesis, Russian Academy of Sciences, 29 Leninskiy Prospekt, 119991 Moscow, Russia; zotkin@ips.ac.ru; 2 Chemistry Department, M.V. Lomonosov Moscow State University, Leninskye Gory 1, 3, 119991 Moscow, Russia; zaitsev@org.chem.msu.ru

**Keywords:** microporous materials, linear rigid-chain polymers, carbocyclic moieties, membrane gas separation

## Abstract

Microporous soluble polymers attract great attention as materials for membrane gas separation, gas storage and transportation, as sorbents, supports for catalysts, and matrices for mixed matrix membranes. The key problems in the development of this area of polymer chemistry include the search for methods of controlling the porous structure parameters and ensuring the stability of their properties over time. In this connection, a fruitful approach is to introduce bulky and rigid, often framework carbocyclic moieties into the polymer backbones and side chains. This review discusses the effect of carbocyclic moieties on gas transport properties, BET surface area, and FFV of glassy polymers, such as polyacetylenes, polynorbornenes, polyimides, and ladder and partially ladder polymers. In the majority of cases, the incorporation of carbocyclic moieties makes it possible to controllably increase these three parameters. Carbocyclic moieties can also improve CO_2_/gas separation selectivity, which is displayed for ladder polymers and polynorbornenes.

## 1. Introduction

Microporous materials, i.e., materials with pores less than 2 nm in size, which is comparable with the diameter of gas molecules, are of great interest to researchers because they can be used for gas storage and for membrane gas and ion separation [1,2,3]. The best known examples of microporous materials are activated carbons, zeolites, metal–organic and covalent–organic frameworks (MOFs and COFs, respectively) [4], as well as organic glassy polymers of intrinsic microporosity [1]. The latter are of interest because, unlike the materials described above, they can be soluble and thus processable, and their mechanical properties can be good enough to form solid articles (fibers and films), which is an essential requirement for fabrication of membranes. The intrinsic microporosity in glassy polymers can be defined as a network of interconnected intermolecular voids less than 2 nm in diameter, which are formed due to loose packing of rigid polymer chains [5]. Since, from a macroscopic point of view, these intermolecular voids cannot be treated as individual phases, the concepts of internal microporosity and free volume are practically indistinguishable, and the microporous structure of microporous polymers can be described using both sorption methods and free volume methods. The most widely used methods of investigation of glassy microporous polymers include the fractional free volume (FFV), positron annihilation lifetime spectroscopy, and low-temperature nitrogen adsorption/desorption. The latter method allows one to evaluate the specific surface area available for nitrogen molecules using polymolecular adsorption theory and Brunauer–Emmett–Teller (BET) equation. Although this method was initially intended to describe a surface having a distinct solid–gas interface, it is also applicable for the intrinsic microporosity. In the case of microporous glassy polymers, BET specific surface area characterizes rather the ability of the polymer to uptake nitrogen in intermolecular voids. Nevertheless, this value well correlates with the free volume of microporous polymers, and the nitrogen adsorption/desorption method is comparatively technically simple. Therefore, this method remains a useful tool to compare different microporous polymers [6]. One more important parameter is gas permeability. For glassy polymers, it usually correlates with the free volume, which is also a practically valuable characteristic of materials.

In recent decades a large number of classes of microporous polymers have been discovered, including partially ladder aromatic polyethers, frequently called PIMs (polymers of intrinsic microporosity) [5,7], ladder Tröger’s base (TB) polymers [8], polyacetylenes (PAs) [9], polybenzoxazoles resulting from thermal rearrangement of polyimides (PIs) and therefore termed thermally rearranged (TR) polymers [10,11], vinyl-addition polynorbornenes (APNBs) [12], polynaphthoylenebenzimidazoles [13], etc. What all these polymers have in common is that they have highly rigid backbones, a linear topology, and are, in most cases, soluble in organic solvents. Microporosity in them is generated during solvent molding or directly in the synthesis, without any additional processing of polymers.

It is important to find methods for controlling microporosity in glassy polymers. There are two main approaches aimed at increasing microporosity in polymers, and both of them involve regulation of the backbone rigidity [14]. One strategy is to restrict backbone rotation through the insertion of multiple bonds or the incorporation of cyclic fragments. The other assumes the introduction of bulky substituents into side chains. The most pronounced effect is obtained when both approaches are combined. The introduction of bulky substituents into side chains of initially rigid-chain polymers leads to a parallel increase in the free volume, specific surface area of micropores, and gas permeability to very high values. In particular, these characteristics of PAs and PNBs bearing trimethylsilyl side groups are much higher than those of related unsubstituted polymers [9,12]. As a result, SiMe_3_-substituted rigid-chain polymers are characterized by record-high gas permeability coefficients, well-developed surfaces with specific surface areas of more than 500 m^2^ g^−1^ due to the large contribution of micropores, and high FFV values exceeding 20% [15,16,17,18]. However, the strategy based on the introduction of SiMe_3_ groups has certain disadvantages, such as the complexity and high cost of the synthesis of monomers, which is often carried out using organomagnesium compounds and toxic chlorosilanes [19]. In addition, the applicability of this method is limited to the maximum number of trimethylsilyl groups per monomer unit and depends on the rigidity of the bonds between the backbones and side-chain substituents. Namely, the longer the spacer between the backbone and the SiMe_3_ group, the less pronounced the effect [20]. In addition, it is difficult to introduce multiple trimethylsilyl groups rigidly bound to the backbone into the side chain; e.g., at most two SiMe_3_ groups can be attached to APNBs [12]. Therefore, the development of alternative methods for controlling the microporosity in glassy polymers remains topical despite the advantages of the trimethylsilyl groups.

One more promising approach involves the incorporation of carbocyclic moieties, including aliphatic and aromatic carbocycles, as well as polycyclic frameworks, into the polymer structure. By combining the structural rigidity and large size of these structural units, one can increase the rigidity of polymer chains and, as a consequence, the free volume, specific surface area, and gas permeability. There are diverse carbocyclic moieties that can be incorporated into both the backbone and side chain.

Up to date, a great amount of polymers containing carbocyclic moieties in their structures have been studied as promising microporous materials, but the relationships between the nature of these moieties and properties of these polymers, such as gas transport properties and specific surface area, have not yet been discussed systematically for a wide variety of polymer structures. The aim of this review is to summarize the advantages in the development of microporous materials based on glassy polymers bearing carbocyclic moieties and to assess their impact on the FFV values and on the sorption and gas transport properties of polymers. In the text below, we will consider how the carbocyclic moieties incorporated into the structure of different classes of polymers, such as PAs, PNBs, PIs, ladder, and partially ladder polymers, influence the properties in question.

## 2. Polyacetylenes

**Scheme 1:**
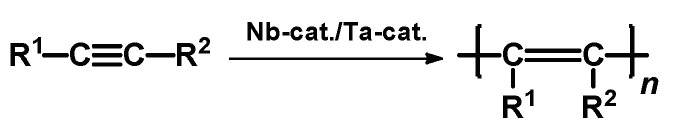
Synthesis of polyacetylenes by polymerization of substituted acetylenes.

Polyacetylenes (PAs) are a large class of polymers whose applications are governed by their rigid-chain structure. The multiple polymerization techniques allow the same alkyne to be used for the synthesis of polymers with different backbone structures [21]. However, most often PAs are conjugated polyene chains formed in the polymerization of substituted acetylenes in the presence of niobium and tantalum chlorides (Scheme 1).

Polyacetylenes bearing bulky substituents exhibit hindered rotation and are therefore rigid-chain polymers. This underlies a loose packing of polymer chains, a large free volume, and, as a consequence, high gas permeability and microporous structure. However, the high gas permeability of PAs is mitigated by low selectivity for gas separation. Moreover, like most large free-volume polymers, PAs undergo intense relaxation aging, viz.; the polymer chains relax with time, which leads to a decrease in the free volume. The decrease in gas permeability due to relaxation aging is most pronounced in thin membranes [22]. This effect can be compensated for by using various methods. In general, there are two approaches to reducing physical aging. The first is intentional acceleration of aging, which can be realized by thermal annealing. It significantly reduces gas permeability (up to ten times) but makes the gas transport properties stable over time, which is important for potential industrial application [23]. The second is the modification of a polymer membrane that makes the polymer more stable to physical aging, e.g., cross-linking [24] or incorporation of additives, such as rigid porous fillers [25] and/or branched polymers (e.g., polyethyleneimine) [26]. The latter approach reduces aging by 2–3 times without significant losses in free volume and gas permeability. In addition to physical aging, PAs also undergo chemical aging (oxidation of the double bonds in the backbone) and sorption aging (the capture of impurities that can block free volume).

The gas transport properties of numerous PAs bearing various substituents, including carbocyclic ones, have been intensively studied for decades (Table 1). Poly(1-trimethylsilylpropyne-1) (PTMSP), invented in 1983, exhibits record-high gas permeability (*P*(O_2_) = 8300 barrer [15]) and a large specific surface area (*S*_BET_ = 780 m^2^ g^−1^ [16]). This compound is often used as a reference, highly permeable polymeric material. The increase in gas permeability upon introducing a bulky, rigid SiMe_3_ group is also observed for many other classes of polymers, e.g., PNBs [27]. The gas permeability and the FFV value of PTMSP and related high-FFV polymers depend strongly on the polymerization conditions (the catalyst, solvent, and temperature influence the microstructure of the resulting polymer) and on the measuring intervals (relaxation aging leads to a rapid decrease in these parameters over time). As a result, experimental data for the polymers in question can differ significantly.

Replacement of the SiMe_3_ group by other fragments usually leads to a decrease in FFV and gas permeability. The only exception is poly(4-methylpentyne-2) [28], which is superior to many PAs in gas permeability, although it remains much below PTMSP. Monosubstituted Pas, including those bearing a bulky substituent, e.g., a *tert*-butyl group [29], usually do not have high FFV values. By and large, the effect of carbocyclic side substituents in PAs is similar to that of the SiMe_3_ group, except that it is less pronounced. The gas transport properties of PAs bearing various carbocyclic substituents are collected in Table 1. One carbocyclic substituent at the double bond of a PA (e.g., poly(1-phenyl-1-propyne)) is insufficient to generate microporosity [20]. However, PAs bearing two carbocyclic substituents bound to the backbone are characterized by high gas permeability. The highest gas permeability was observed for PAs with the fluorene and phenanthrene substituents [30].

**Table 1:** Gas transport properties and fractional free volumes of polyacetylenes ^a^.

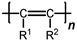		*P*, Barrer ^b^				α_i/j_		FFV, %	Ref.
R^1^	R^2^	N_2_	O_2_	CO_2_	CH_4_	O_2_/N_2_	CO_2_/N_2_		
Me	SiMe_3_	4900	8300	–	–	1.7	–	26 – 34	[15]
Me	SiMe_3_	12,000	15,000	47,000	30,000	1.3	4.1		[31]
Me	SiMe_3_	6700	10,000	33,000	16,000	1.5	4.9		[32]
Me	SiMe_3_	9400	13,000	44,000	23,000	1.4	4.7		[22]
Me	*i*-Pr	1300	2700	11,000	2900	2.0	8.1	28	[28]
Ph	Me	2.2	6.3	25	2.8	2.9	11	22	[20]
Ph	Et	4.5	12	40	4.4	2.7	8.9	–	[20]
Ph	*н*-C_6_H_13_	5.5	14	48	14	2.6	8.7	15	[20]
Ph	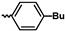	60	100	450	120	1.7	7.5	–	[33]
Ph	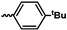	500	1100	4800	1400	2.2	9.6	–	[33]
Ph	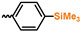	560	1200	4900	1600	2.1	8.8	26	[34]
Ph	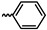	410	910	–	–	2.2	–	23	[34]
Ph	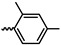	290	530	2000	780	1.8	6.9	24	[35]
Ph	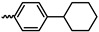	83	230	1300	240	2.8	15	21	[36]
Ph	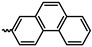	650	1300	5300	1500	2.0	8.2	–	[30]
Ph	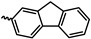	1300	2200	8500	2400	1.7	6.5	–	[30]
Ph	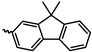	3300	4800	17,000	9100	1.5	5.2	–	[37]
Ph	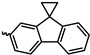	300	660	3200	810	2.2	11	–	[37]

^a^ Hereinafter the permeability coefficients are presented for pure gas experiments, room temperature, and pressure of 1 atm. ^b^ 1 barrer = 10^−10^ cm^3^(STP) cm cm^−2^ s^−1^ cmHg^−1^.

**Scheme 2:**
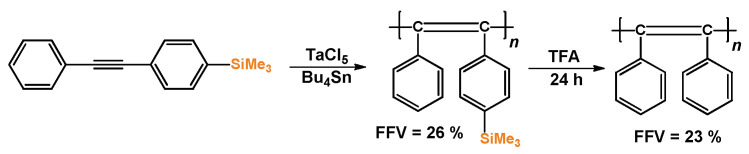
Synthesis of poly(diphenylacetylene) [38].

Certain PAs with two carbocyclic substituents, e.g., poly(diphenylacetylene), are insoluble polymers. Studies of such systems involve preliminary fabrication of films of soluble trimethylsilyl-substituted PAs followed by desilylation with trifluoroacetic acid (Scheme 2). As a result, the gas permeability coefficients and FFV values most often decrease (for example, the FFV of poly(1-phenyl-2-(*p*-trimethylsilyl)phenylacetylene) reduces from 26% to 23%, and *P*(O_2_) reduces from 450 to 86 barrer after desilylation), although the reverse trend was also reported [35].

Among PAs bearing carbocyclic substituents, indane-containing polymers are characterized by the highest gas permeability coefficients (*P*(O_2_) up to 19,000 barrer) [31]. In certain cases, these polymers exhibit gas permeability coefficients that surpass those of PTMSP, as shown in Table 2. In addition, indane-containing PAs are less prone to physical aging: e.g., the polymer bearing the 1,1,3,3-tetramethylindane and phenyl substituents has the gas permeability coefficient *P*(O_2_) = 14,000 barrer, which decreases by only 29% after 90 days. These polymers are very soluble in organic solvents and exhibit high thermal stability (decomposition temperatures are above 400 °C).

**Scheme 3:**
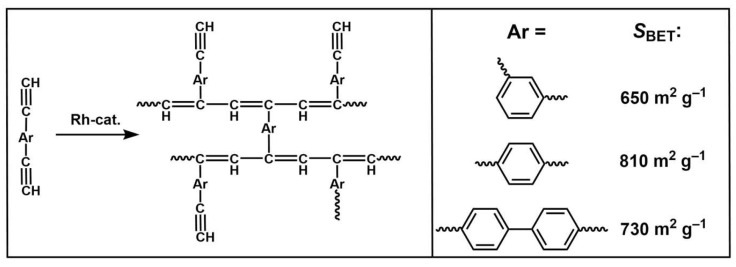
Synthesis and BET surface areas of branched polyacetylenes.

The synthesis of cross-linked PAs with carbocyclic substituents is also of interest, because these polymers are less prone to aging. A series of branched PAs were synthesized on rhodium catalysts [40], and their sorption properties were studied. The polymers display BET surface areas in the range of 650–730 m^2^ g^−1^ (Scheme 3) and micropore volumes of 0.21–0.25 cm^3^ g^−1^. These polymers are analogues of poly(divinylbenzenes) but have a microporous structure due to the presence of double bonds in the backbone. The aryl linker influences the specific surface area of the resulting polymer. The largest specific surface area was obtained for the polymer with the *para*-attached monophenyl linker.

In conclusion, PAs bearing carbocyclic moieties in side chains are highly permeable polymers characterized by high FFV values. Certain PAs are comparable with PTMSP in these parameters. Significant disadvantages of PAs include low selectivity for gas separation and rapid relaxation aging and thus make the polymers of limited use. Nevertheless, there are examples of not-so-highly permeable PAs containing carbocyclic moieties with reasonable CO_2_/N_2_ separation selectivity (up to 13), i.e., PAs bearing indane substituents without methyl groups.

## 3. Polynorbornenes

**Scheme 4:**
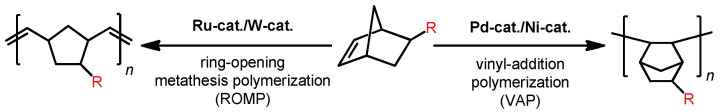
Synthesis of polynorbornenes.

Polynorbornenes (PNBs) form one more abundant class of polymers. Many PNBs have a microporous structure, a large specific surface area [6], and high gas permeability coefficients [41]. For example, the BET surface area of SiMe_3_-substituted vinyl-addition PNBs achieves 790 m^2^ g^−1^, and *P*(CO_2_) is up to 20,000 barrer. For the majority of PNBs, gas permeability changes in a sequence: *P*(N_2_) < *P*(CH_4_) < *P*(O_2_) < *P*(CO_2_). Herewith, *P*(He) and *P*(H_2_) can be both less and greater than *P*(CO_2_). A salient feature of PNBs is the possibility to synthesize fundamentally different polymers from the same monomer using two different kinds of polymerization, viz., metathesis and vinyl-addition polymerization (Scheme 4). Both types of PNBs include microporous polymers. The backbone of metathesis PNBs (MPNBs) contains rigid five-membered rings, while that of vinyl-addition PNBs (APNBs) contains norbornyl rings. This provides a loose packing of polymer chains and influences the FFV value and, as a consequence, the sorption and gas transport properties. In most cases, the gas permeability coefficients of APNBs are 1–2 orders of magnitude higher than those of MPNBs, the specific surface area and, as a consequence, the free volume of the former being also larger [12] due to the increased backbone rigidity in APNBs. Nevertheless, simple synthesis of MPNBs makes them convenient for studying structure–property correlations. It is worth noting that the correlations established for MPNBs allow one to predict the properties of APNBs.

Side-chain substituents have a strong impact on the properties of PNBs. Like in the case of PAs, the introduction of the SiMe_3_ group into the side chain of PNBs causes a large increase in the gas permeability coefficient (by about two orders of magnitude) and specific surface area (Table 3) [41]. Replacement of the trimethylsilyl group by carbocyclic substituents in the side chain of PNBs also leads to an increase in the gas permeability coefficient, specific surface area, and FFV value (Table 3); however, the effect is less pronounced and sensitive to the size and nature of the carbocycle. The specific surface area and gas permeability coefficients increase to a greater extent as the size of the carbocycle increases and as bridging fragments are added to make the carbocycle more rigid.

As a result, an APNB bearing the norbornyl substituent is characterized by the gas permeability coefficient *P*(O_2_) = 1100 barrer and a specific surface area of 620 m^2^ g^−1^ [42,43]. In contrast to the increase in the specific surface area, the increase in gas permeability depends more strongly on the rigidity of the carbocycle than on the size of the carbocycle as the critical parameter, viz., the APNB with the rigid cyclopropyl substituent obtained by cyclopropanation of poly(5-vinyl-2-norbornene), is characterized by higher gas permeability than the APNB with a bulkier, although more flexible, cyclohexyl substituent [44].

Interestingly, structure modification of PNBs by introducing carbocyclic substituents not only increases the gas permeability but in some cases improves the selectivity for separation of CO_2_-containing gas pairs, which was not observed upon introduction of the SiMe_3_ group. For instance, the gas permeability for CO_2_ and the selectivity for separation of CO_2_/N_2_ mixture of the APNBs bearing the norbornyl substituent are above the upper bound in the 2008 Robeson plot. It should be noted that the exponential dependence of the diffusion coefficient and solubility on the squared effective diameter of the penetrant gas molecule and on the potential of the gas–polymer interaction, respectively, is retained, which suggests the absence of specific interaction [43].

**Table 3:** Gas transport properties and BET surface areas of PNBs bearing trimethylsilyl and carbocyclic side groups.

Polymer	*P*, Barrer				α_i/j_		*S*_BET_, m^2^ g^−1^	FFV, %	Ref.
	N_2_	O_2_	CO_2_	CH_4_	O_2_/N_2_	CO_2_/N_2_			
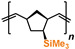	7.1	28	120	17	3.9	17	–	–	[45]
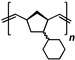	1.4	4.9	18	2.6	3.5	13	<10	18	[42,43]
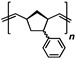	0.8	2.7	14	0.82	3.4	18	–	–	[43]
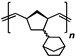	–	3.8	29	4.0	–	–	<10	–	[42,43]
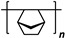	1.5	6.9	34	2.6	4.5	23	–	–	[27]
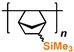	300	780	4400	790	2.6	15	–	–	[27]
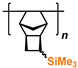	430	1100	6000	1100	2.5	14	–	–	[46]
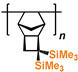	2700	4800	20,000	6900	1.8	7.4	790	–	[17]
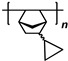	22	70	510	44	3.2	23	420	20	[44]
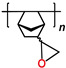	3.8	7.6	120	7.1	2.0	32	56	9	[44]
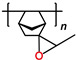	15	50	1000	20	3.3	67	480	27	[47]
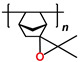	200	650	5400	300	3.3	27	600	33	[47]
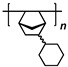	14	50	360	24	3.6	26	480	19	[42,43]
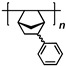	13	41	450	25	3.2	35	48	–	[42,43]
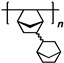	27	110	1100	79	4.1	41	620	–	[42,43]

The incorporation of oxygen-containing cyclic fragments into PNB side chains is at least as promising a structural modification aimed at improving the gas transport properties of polymers, e.g., gas permeability and CO_2_/gas selectivity. Thereby, the incorporation of these moieties makes it possible to design high-performance membranes for CO_2_ capture. For instance, recently obtained APNBs bearing epoxy moieties in side chains combine high gas permeability with remarkable selectivities for separation of CO_2_ from binary gas mixtures. The gas permeability coefficients of an epoxidized APNB based on 5-ethylidene-2-norbornene are above the upper bound in the 2019 Robeson plot for the CO_2_/N_2_ pair. Moreover, the gas permeability of this polymer remains almost unchanged over a period of 100 days [47]. Interestingly, the effect of the oxirane ring is more pronounced when it forms a spiro structure with the cyclic fragment of the monomer unit of the polymer rather than when it is simply present as a side-chain substituent (Table 3). It follows that the effect is sensitive to the possibility of rotation of the oxirane ring. Probably, a closer packing of polymer chains is possible provided that the oxirane ring can rotate. This also influences the specific surface area, which can be as large as 600 m^2^ g^−1^ for the polymers with the spiro-epoxy moiety. In contrast to the PNBs with carbocyclic substituents, specific interactions between CO_2_ molecules and the polymer matrix occur in the epoxidized polymers [47].

**Figure 1:**
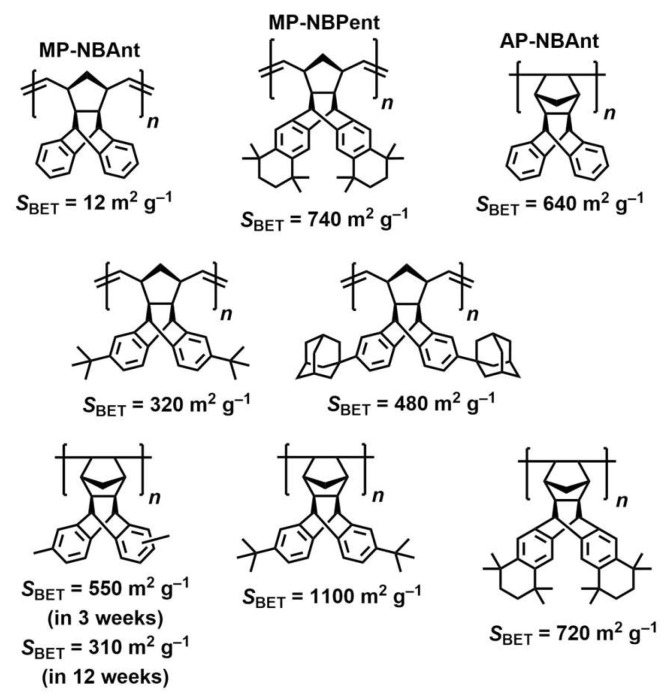
BET surface areas of PNBs bearing 9,10-dihydroanthracene side substituents. The images of the structures were reproduced with permissions from [43,49].

The incorporation of 9,10-dihydroanthracene fragments, including substituted ones, into the side chains of PNBs belongs to the most interesting methods for improving the BET surface areas and gas permeability of these polymers. This can be simply done in the step of monomer synthesis using the Diels–Alder reaction of norbornadiene-2,5 with anthracene derivatives [48]. For both MPNBs and APNBs, the incorporation of 9,10-dihydroanthracene moieties leads to the formation of microporous structures and to large specific surface areas (Figure 1) that differ depending on the presence and volume of substituents. If the 9,10-dihydroanthracene fragments bear bulky substituents, such as *tert*-butyl, adamantyl groups, and additional cyclic fragments, the specific surface area can be as large as 740 m^2^ g^−1^ for MPNBs [42] and 1100 m^2^ g^−1^ for APNBs [49]. These are the record-high values for linear PNBs bearing substituents comparable with the monomer unit in size. For certain MPNBs, the specific surface area values correlate with the volumes of substituents and with the rigidity of their bond with the 9,10-dihydroanthracene unit. First, in the case of the adamantyl substituent, the specific surface area is larger than in the case of the *tert*-butyl one, and second, the specific surface area of the polymer bearing the rigid pentacyclic core is even larger than that of the polymer bearing the bulkier adamantyl substituents. No explicit correlations were demonstrated by APNBs; however, their specific surface areas are by and large much larger than those of MPNBs bearing the same substituents due to the higher rigidity of their backbones [42,49]. Like other microporous polymers, PNBs undergo relaxation aging, viz., the specific surface area of an APNB bearing a substituent with the 9,10-dihydroanthracene fragment and two Me groups decreased by a factor of 1.8 within 9 weeks [50].

At present, there are only three cases to exemplify the gas permeability and fractional free volume studies of the PNBs bearing 9,10-dihydroanthracene moieties (Table 4). Nevertheless, the available data suggest that the introduction of substituted 9,10-dihydroanthracene fragments also leads to a significant increase in gas permeability coefficients and FFV values. The gas permeability coefficients increase upon the introduction of pentacyclic cores into the side chains of MPNBs to an even greater extent than upon the introduction of three trimethylsilyl groups per monomer unit [43]. It should be noted that the polycyclic side substituents in the polymers presented in Table 4 are rigidly bound to the cyclic moieties in the monomer units and that rotation about the C–C bond is impossible. Higher BET surface areas and gas permeability coefficients of these polymers as compared to those of the PNBs bearing carbocyclic substituents bound to the cyclic moieties in the monomer units through the single C–C bond confirm that restriction of rotation of the carbocycle is at least equally important as its volume.

5,6-norbornene dicarboximides (nadimides) also demonstrate an increase in FFV values and gas permeability upon the introduction of carbocyclic substituents (Table 5). The best results are obtained when the polymer structure is modified by simultaneously introducing carbocycles and some other functionalities, e.g., CF_3_ groups. For example, an APNB based on nadimide bearing a phenyl substituent with two *meta*-CF_3_ groups is characterized by high gas permeability and selectivity for gas separation (*P*(CO_2_) = 1400 barrer, α(CO_2_/N_2_) = 33).

**Figure 2:**
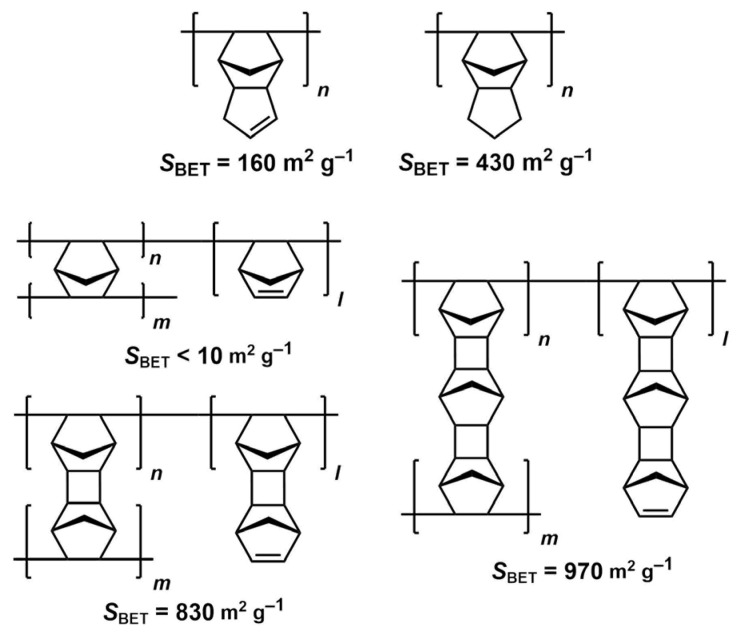
BET surface areas of APNBs based on dicyclopentadiene, as well as norbornadiene-2,5 and its oligomers. The figure was reproduced with permissions from [55,56].

Microporous PNBs bearing carbocyclic substituents can also be prepared from commercially available reagents without using complicated multi-stage syntheses of monomers. Addition polymers based on dicyclopentadiene were synthesized [55], and their BET surface areas were studied (Figure 2). The polymers have a large specific surface area (up to 430 m^2^ g^−1^) due to the presence of the five-membered ring rigidly bound to the backbone and playing the role of the side group. Interestingly, hydrogenation causes a nearly three-fold increase in the specific surface area. The BET surface areas of the APNBs based on norbornadiene-2,5 and its oligomers (Figure 2) were also studied [56]. It was found that the specific surface area regularly increases on going from the polymer based on norbornadiene-2,5 to that based on the trimer of norbornadiene-2,5, namely, if the parent polymer is not microporous (*S*_BET_ < 10 m^2^ g^−1^), the polymers based on the dimer and trimer are characterized by rather large specific surface areas of 830 and 970 m^2^ g^−1^, respectively. All these polymers have branched or cross-linked structures, are insoluble in organic solvents, and, therefore, can be used only as adsorbents rather than polymeric membranes.

So-called polymers with side chain porosity [57,58] form a particular subclass of PNBs. They are obtained in two stages by Diels–Alder homopolyaddition of substituted anthracenenorbornene, affording an oligomeric product containing the bicyclic norbornene moiety as an end group, followed by ring-opening metathesis polymerization of the macromonomer. The resulting polymers contain the metathesis PNB backbone and long, rigid polycyclic side substituents and can be treated as polymer brushes. Being, in essence, the metathesis PNBs, polymers with side chain porosity are microporous polymers characterized by a large specific surface area (up to 780 m^2^ g^−1^) and high gas permeability coefficients (Table 6). Interestingly, the macromonomers formed in the first stage of synthesis are not microporous materials, while the microporous structure is generated in the course of metathesis polymerization. A study [59] of the effect of carbocyclic side chain length (and the macromonomer chain length) on the properties of polymers revealed that both specific surface area and gas permeability increase with increasing chain length. The nature of the substituents R (Table 6) also significantly influences the properties of the polymers. The largest specific surface area and the highest gas permeability were obtained for the polymers modified with CF_3_ groups.

Thus, PNBs provide excellent examples of high gas permeability and large specific surface area polymers prepared by introducing rigid carbocyclic moieties into side chains. In some cases, the polymers thus obtained are superior to related PNBs bearing trimethylsilyl groups in properties. The effect of the carbocyclic substituents depends on their volume, structural rigidity, the rigidity of their bond with the backbone, and the presence of additional substituents that can provide a synergistic effect.

## 4. Polyimides and Related Polymers

Polyimides (PIs) are obtained by polyaddition of dianhydrides and aromatic diamines followed by intramolecular cyclization of poly(amidocarboxylic acid) (PAA). Owing to the presence of rigid imide rings, PIs are also prone to the formation of microporous structures. Polyimides possess valuable performance properties including increased thermal stability (decomposition temperatures are above 400 °C), chemical stability, and good mechanical properties. They also enable fabrication of films and fibers in the PAA step. However, the gas permeability of conventional polyimides is low, and the specific surface area is small due to the presence of polar imide groups. For example, polyimide based on *p*-phenylenediamine and 4,4′-(hexafluoroisopropylidene)diphthalic anhydride is characterized by *P*(O_2_) of 4.2 barrer and is not a microporous polymer. The FFV values can be increased and the sorption and gas transport properties improved by, e.g., incorporating carbocyclic moieties into the backbone and side chains of PIs. This can be done by introducing additional carbocycles into the structure of diamines and dianhydrides (Table 7). The best results are obtained using dianhydrides with polycyclic frameworks containing no rotatable C–C bonds.

To date, a large number of PIs of different structure have been synthesized and studied [68]. The most widely used dianhydrides include pyromellitic dianhydride (PMDA), 3,3′,4,4′-biphenyltetracarboxylic dianhydride (BPDA), and a dianhydride based on 2,2-bis(3,4-dicarboxyphenyl)hexafluoropropane (6FDA) [69]. The last-mentioned compound containing two bulky CF_3_ groups, which strongly restrict rotation about the single C–C bonds, appeared to be rather promising for achieving the above goals. Koros [70] and Okamoto [60] were among the first researchers who synthesized various PIs based on 6FDA and studied their gas transport properties. The role of diamines is played by similar structures consisting of phenyl [60], dinaphthyl [66], indane [61,62], etc., fragments.

The gas transport properties and BET surface areas of PIs were additionally improved upon incorporating even bulkier and more rigid blocks, such as spirobifluorene [64], spirobisindane [65], triptycene [66], and ethanoanthracene units [67] into the polymer backbone (Table 7). For instance, a PI synthesized using a dianhydride based on ethanoanthracene and a diamine based on substituted dinaphthalene, DMN-EA, exhibits a record-high (for PIs) gas permeability (*P*(O_2_) = 1400 barrer) and a large specific surface area (*S*_BET_ = 620 m^2^ g^−1^). A similar effect was observed upon structure modification with triptycene units; namely, the specific surface area reaches a value of 430 m^2^ g^−1^ for PIs based on triptycene-containing diamines [71] and 760 m^2^ g^−1^ for PIs based on triptycene-containing dianhydrides [66]. Branched triptycene-containing PIs also have a large specific surface area (up to 740 m^2^ g^−1^) [72]. Like for PIMs, the incorporation of spirocyclic units into the PI backbone is a powerful method for the generation of microporosity.

**Scheme 5:**
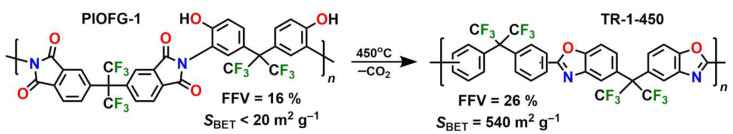
Thermal rearrangement of PIOFG-1 affording TR-1-450 [10].

Thermal rearrangement of polyimides to polybenzoxazoles and polybenzothiazoles is one more approach to create microporosity. Heat treatment of PIs with *ortho*-functional groups (usually OH and SH) relative to the imide bond (so-called PIOFG polymers) leads to intramolecular cyclization of the backbone with the formation of new heterocyclic compounds, e.g., benzoxazoles (Scheme 5). This is followed by spatial rearrangement of polymer chains and formation of micropores. The resulting polymers are also called thermally rearranged (TR) polymers. Compared to the starting PIs, the free volume in TR polymers is nearly doubled, and the specific surface area is also much larger [10]. The heat treatment conditions (usually 350–450 °C) influence the conversion and, as a consequence, the properties of the synthesized polymers. For example, the polymer PIOFG-1 treated at 450 °C is characterized by high gas permeability (*P*(CO_2_) = 1600 barrer), high selectivity (α(CO_2_/CH_4_) = 50), and a developed surface (specific surface area is 510 m^2^ g^−1^) [73]. Moreover, TR polymers show almost no plasticization with CO_2_, which leads to a considerable decrease in the selectivity for gas separation [74]. Taking into account the advantages mentioned above, TR polymers remain at the focus of intensive research [75].

**Figure 3:**
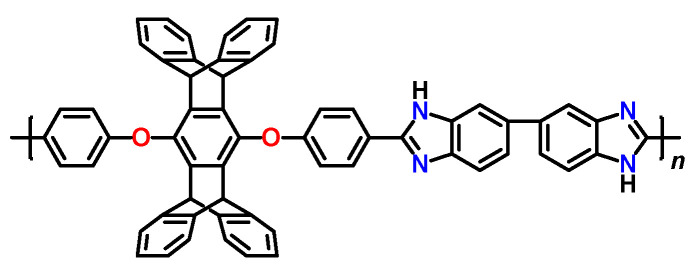
Structure of pentiptycene-based polybenzimidazole.

The closest analogues to polybenzoxazoles are polybenzimidazoles. These polymers represent a class of heterocyclic polymers containing benzimidazole fragments in main chains. They are synthesized via high-temperature polycondensation between aromatic tetramine and dicarboxylic acid accompanied by intramolecular cyclization. A valuable feature of polybenzimidazoles is extremely high thermal resistance. Herewith, they are usually nonporous and are characterized by low gas permeability, especially at low temperatures. For example, the N_2_ permeability of *m*-PBI is evaluated as 0.0025 barrer at 3 atm and 35 °C [76]. There are a number of examples of the incorporation of carbocyclic moieties into their main chains as a way to improve their sorption and gas transport characteristics. Kumar et al. [77] synthesized a series of copolymers containing 5–10% units with carbocyclic moieties (dicarboxyl [2.2]paracyclophane or spirobisindane moieties), which showed enhanced CO_2_ sorption. Recently, polybenzimidazole containing polycyclic (pentiptycene) moieties in each monomer unit (Figure 3) has been developed [78]. This polymer turned out to be perspective material for high-temperature H_2_/CO_2_ separation. Acid-doped film from this polymer has exhibited 500% higher H_2_ permeability and 230% higher H_2_/CO_2_ selectivity at 180 °C in comparison to *m*-PBI.

Thus, polyimides and related heterocyclic polymers are a long-studied, important class of polymeric materials for membrane gas separation. Incorporation of rigid polycyclic blocks into the backbone improves the gas transport characteristics and favors generation of high porosity.

## 5. Polymers of Intrinsic Microporosity (PIMs)

**Scheme 6:**
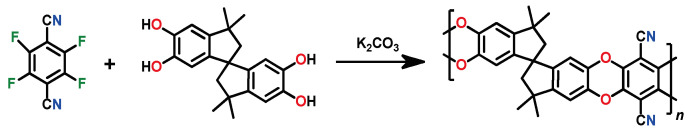
Synthesis of PIM-1.

So-called polymers of intrinsic microporosity (PIMs) deserve particular attention. Although many polymers described above also possess intrinsic microporosity, the term PIMs is traditionally applied to partially ladder aromatic polyethers with rigid, non-rotatable backbones. Interestingly, these polymers simultaneously have a high gas permeability and selectivity. The first PIM (PIM-1 polymer) was obtained in 2004 [79] by double polycondensation of 5,5′,6,6′-terahydroxy-3,3,3′,3′-tetramethyl-1,1′-spirobisindane and 1,4-dicyanotetrafluorobenzene (Scheme 6). Owing to the presence of a spirocyclic unit, the polymer has a high FFV value (24–26%), a large specific surface area (*S*_BET_ = 850 m^2^ g^−1^) [79], and a high gas permeability (*P*(O_2_) = 2300 barrer) [80]. PIM-1, a yellow fluorescent glassy polymer with a decomposition temperature above 350 °C, is soluble in most conventional organic solvents.

The «first-generation» PIMs contain rigid spirocyclic units in the backbone and can be obtained by polycondensation of a tetrahydroxy aromatic compound with an aromatic compound containing four halogen (usually fluorine) atoms by the mechanism of nucleophilic aromatic substitution in the presence of bases. In addition, PIMs based on the rigid triptycene [81] and dibenzomethanopentacene [82] structural units playing the role of spirocyclic fragment have been reported. These PIMs are true ladder polymers. Many PIMs are characterized by large specific surface areas and high gas permeability coefficients (Table 8). Their FFV values can be very high, e.g., 31% for the triptycene-based PIM-TMN-Trip and 28% for the tetramethylnaphthalene-based PIM-TMN-SBI [81]. The most interesting gas transport characteristics from the standpoint of separation of CO_2_/N_2_ gas mixtures are demonstrated by the PIMs with ladder polycyclic moieties incorporated into the backbone, viz., PIM-TMN-Trip and copolymers, with the PIM-DBMP units. The homopolymer PIM-DBMP is insoluble in organic solvents; however, its copolymers with PIM-1 and a polymer modified using a polymeranalogous reaction with NH_2_OH (PIM-DBMP-AO) are soluble, thus being suitable for film fabrication. Compared to PIM-1, the copolymers are characterized by higher gas permeability for CO_2_ and selectivity for separation of CO_2_/N_2_ and CO_2_/CH_4_ mixtures. The best combination of the gas transport characteristics was achieved at a PIM-1:PIM-DBMP ratio of 1:1. The incorporation of adamantyl-containing units instead of spiro units into the structure of PIM-1 results in a reduction in gas permeability and a simultaneous increase in gas separation selectivity. As a result, ADM-PIM is characterized by excellent O_2_/N_2_ selectivity, and its data are located above the 2008 upper bound in Robeson’s plot. Herewith, the amount of adamantyl units almost does not influence BET surface area [83]. One more advantage of these copolymers is that they are less prone to aging compared to PIM-1, viz., their gas permeability decreases by 30–50% (cf. 70–80% for PIM-1) over a period of more than 1000 days [82]. The copolymer PIM-DBMP-AO is characterized by a lower gas permeability and a much higher selectivity for separation of binary gas mixtures containing CO_2_ [82]. Moreover, PIMs are integrated with polymers from other classes, e.g., PIs [84]. Based on the aforesaid, this broad class of polymers continues to expand. The properties of PIMs have been thoroughly analyzed in reviews [85,86].

The fully carbocyclic analogs of PIMs are also prospective candidates as materials for gas separation membranes. Zhu et al. [89] reported the synthesis and gas transport properties of OH-functionalized carbon main chain PIMs. These polymers displayed good separation performance for H_2_-containing gas pairs: H_2_/N_2_ and H_2_/CH_4_ separation parameters were located near the 2008 upper bound in Robeson’s plot. Despite the presence of OH groups, one of them (HSBI-Is, Table 8) had reasonable gas permeability and BET surface area. It can be expected that fully carbocyclic PIM analogs not containing OH groups will also have interesting gas separation properties.

Concluding this chapter, PIMs are a large class of partially ladder and ladder polymers characterized by high gas permeability and large specific surface area. The presence of rigid cyclic moieties incorporated into the backbone underlies high porosity and gas permeability.

## 6. Tröger’s Base Polymers

**Scheme 7:**
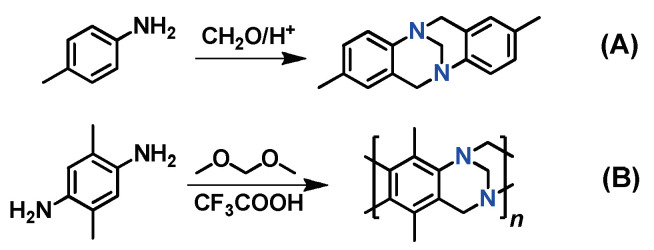
Synthesis of Tröger’s base (**A**); polycondensation affording TB polymers (**B**).

Tröger’s base was for the first time obtained in 1887 by the reaction of *p*-toluidine with formaldehyde in an acidic medium, which led to a tetracyclic structure (Scheme 7A). This reaction is a prototype for polymerization reactions affording Tröger’s base polymers (TB polymers). These polymers can be synthesized using bifunctional compounds. Polycondensation of aromatic diamines with formaldehyde or dimethoxymethane in trifluoroacetic acid results in high-molecular-weight polymers that are soluble in chloroform and are thermally stable at temperatures up to 350 °C (Scheme 7B). The commercial availability of diamines provides an opportunity for a one-step synthesis, which is a significant advantage of TB polymers over other polymer classes, such as PNBs, PAs, and PIMs discussed above, which frequently require the multi-stage synthesis of monomers.

Various TB polymers of different structures have been studied [8] to date. Many of them have large specific surface area and high gas permeability (Table 9). In general, the permeability level of TB polymers is lower in comparison to PIMs and PAs. Nevertheless, they are characterized by high CO_2_/N_2_ and CO_2_/CH_4_ separation selectivity, which makes them suitable candidates for the separation of CO_2_ from “flue” gas or natural gas. One more feature of TB polymers is specific surface area as high as 620–1000 m^2^ g^−1^ comparable to the specific surface area of PIMs. The bicyclic TB unit formed upon condensation has a highly rigid structure and restricts free rotation, which leads to loose packing of polymer chains and formation of micropores. This is most pronounced in the case of ladder polymers with backbones consisting of fused rings. Ladder polymers can have high FFV values, e.g., 28% for PIM-EA-TB [90] and 26% for PIM-MP-TB [91]. The presence of a flexible methylene bridge in the backbone disrupts the ladder structure and, as a consequence, leads to a smaller specific surface area and lower gas permeability. However, insertion of carbocycles, especially adamantyl moieties, into the methylene bridge again causes these parameters to increase [92]. By analogy with PIMs, the appearance of the spirocyclic unit also favors an increase in the specific surface area and gas permeability. The largest specific surface area and the highest gas permeability were obtained for the ladder TB polymers containing rigid ethanoanthracene and triptycene units [93]. For example, triptycene-containing PIM-BTrip-TB has very high gas permeability (*P*(O_2_) = 3300 barrer) and a large specific surface area (*S*_BET_ = 870 m^2^ g^−1^) [94]. Relaxation aging of these polymers causes the gas permeability to be halved within 100 days on average. The ladder structure provides TB polymers for one more feature, i.e., the preservation of porous structure at the incorporation of OH groups. Similarly to PIMs, OH-functionalized TB polymers can also possess reasonable gas permeability and BET surface area up to 430 m^2^ g^−1^ [95].

**Figure 4:**
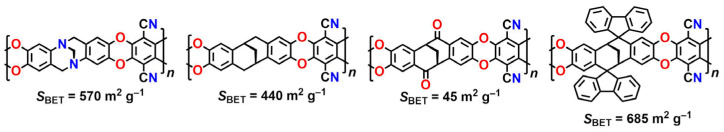
BET surface areas of the carbocyclic analogues of TB polymers.

The presence of nitrogen is not necessary for microporosity of TB polymers. In [99], the porosity of a series of ladder aromatic polyethers (with the main chains similar to those of PIMs) containing TB units or their carbocyclic analogues (Figure 4) was studied. All these polymers, excluding the polymer with carbonyl groups, displayed large BET surface areas. The replacement of nitrogen atoms in TB units by CH groups leads to the reduction in BET surface area, but the replacement of them by spiro fluorene fragments increases *S*_BET_ by 20%. Thus, carbocyclic analogs of TB polymers are also perspective polymers with tunable microporosity.

Thus, microporous polymers of different morphology can be synthesized from readily available reagents in one step by the reaction affording TB. The presence of rigid bicyclic moieties combined with carbocyclic moieties both in the backbone and in the side chains of TB polymers favors high porosity and high gas transport properties.

## 7. CANAL Polymers

**Scheme 8:**
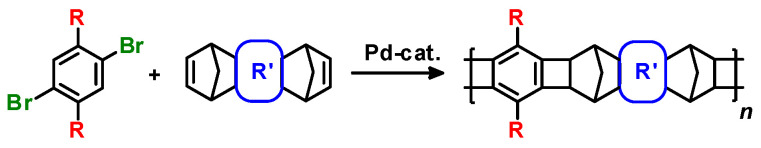
General scheme of CANAL reaction.

**Figure 5:**
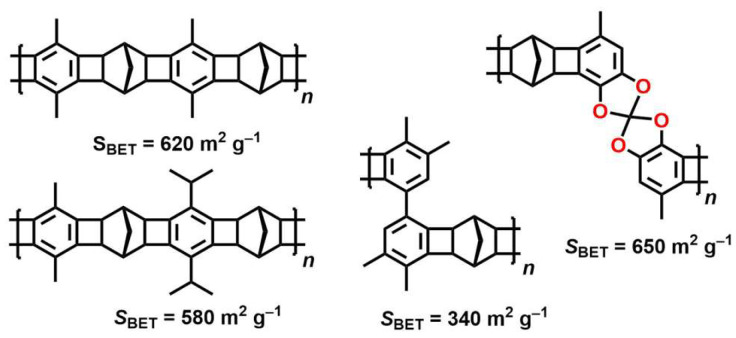
BET surface areas of certain CANAL polymers. The structure images are adapted from [100,102,103].

Ladder polymers obtained by catalytic arene-norbornene annulation (CANAL, Scheme 8) form a particular class of microporous polymers termed CANAL polymers. The CANAL reaction affords very rigid structures, thus being a powerful tool for the design of microporous materials for gas storage and membrane gas separation. Although this type of polycondensation was discovered quite recently, the gas transport properties and BET surface areas of a large number of CANAL polymers have been studied to date (Figure 5, Table 10). The first CANAL polymers synthesized from norbornadiene-2,5 and *p*-dibromobenzene [100,101] had bowing ribbon backbones with in-plane kinks. As in the case of TB polymers, insertion of ordinary C–C bonds into the CANAL polymer backbone disrupts its ladder structure, and, as a consequence, free rotation about these bonds becomes possible. This leads to a closer packing of polymer chains and to a decrease in the specific surface area. On the contrary, incorporation of spirocyclic units into the polymer backbone is an efficient method for increasing the specific surface area of the CANAL polymers [102].

A series of CANAL polymers containing fluorene and dihydrophenanthrene blocks were synthesized [104]. The polymer backbone has a ribbon-like geometry with 2D contortions, which leads to a looser packing of polymer chains. The resulting polymers have a large specific surface area and offer high gas permeability and high selectivity for gas separation (Table 10). The CANAL polymer bearing two methyl groups at the fluorene unit exhibits the highest gas permeability (*P*(O_2_) = 1400 barrer) and the largest specific surface area (*S*_BET_ = 1200 m^2^ g^−1^). Replacement of two methyl groups by a spirocyclic unit causes a decrease in these parameters. The lowest gas permeability coefficient and specific surface area were obtained for the CANAL polymer bearing the dihydrophenanthrene unit, which can be explained by its less rigid structure compared to that of the fluorene moiety.

A study of the aging behavior of the CANAL polymers showed that the gas permeability decreased on average tenfold after 150 days for all gases. The performance of many polymers was above the upper bound in the 2008 Robeson plot for the H_2_/CH_4_ and CO_2_/CH_4_ pairs. The CANAL polymers with spirobifluorene units in the backbone [105] demonstrated lower gas permeability coefficients and had smaller specific surface areas compared to those of the CANAL polymers mentioned above.

Thus, rigid 3D structure allows CANAL polymers to have a large free volume and specific surface area and offer high gas permeability. Simple synthesis of CANAL polymers makes them candidate materials for gas storage and membrane gas separation.

## 8. New Types of Polymers: Combining Different Structural Motifs

**Scheme 9:**
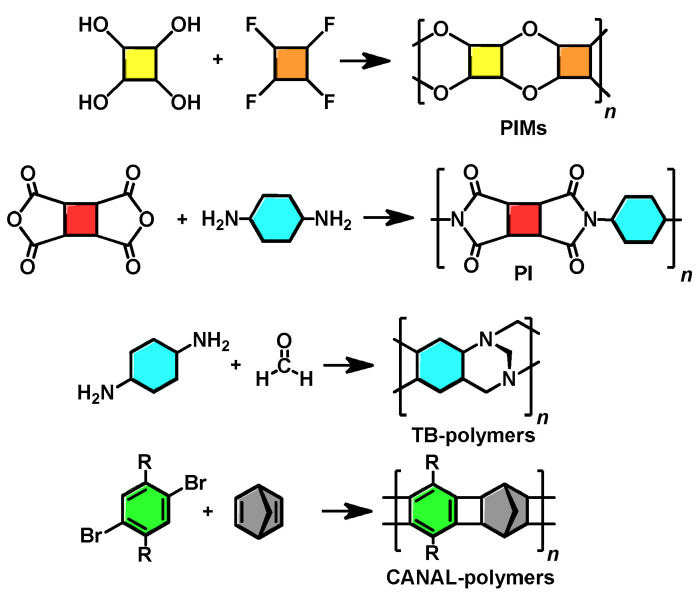
Diverse synthetic routes to microporous polymers.

A wide variety of glassy PIMs were developed in the last two decades. Main families, including PAs, PNBs, PIs, TB polymers, PIMs, and CANAL polymers, were considered above (Scheme 9). Even greater versatility of polymer structures can be obtained by combining different-type structural motifs to give, e.g., PIM-PIs, TB polymers, CANAL-PIs, and many other combinations. The properties of such polymers have been thoroughly analyzed in numerous recent reviews [8,85,106,107].

This approach allows one to synthesize polymers with new properties that are not possessed by the individual constituents. For instance, most PIs have no large specific surface area and do not offer high gas permeability, while high-molecular-weight CANAL polymers with desired mechanical properties are often difficult to synthesize. However, PIs containing repeating units based on CANAL diamines (Table 11) exhibit good mechanical properties and have a large specific surface area (*S*_BET_ in the range of 200–620 m^2^ g^−1^) and high gas permeability (*P*(O_2_) to 390 barrer) [108,109,110]. In this case, the ladder CANAL units are responsible for the generation of microporosity, while PIs provide good mechanical properties.

Among the polymers in question, CANAL-PIs with two methyl groups in *ortho*-positions relative to the imide bonds are characterized by the largest specific surface areas and highest gas permeability coefficients, which can be explained by looser packing of polymer chains due to restricted rotation about the imide bonds. An aging study showed that the gas permeability of the most permeable polymer decreases by less than 30% after 100 days. The CANAL-TB polymers were synthesized [111] by analogy. Their gas permeability and specific surface areas appeared to be even higher than those of CANAL-PIs.

Combining the structures of PIMs and PIs also gave excellent results [84]. A series of triptycene-based PIM-PIs were synthesized [112]. The composition of the polymer backbone was varied by performing polycondensation using different aromatic diamines (Table 12). The specific surface area of certain triptycene-based PIM-PIs can be as large as 840 m^2^ g^−1^, a colossal value for PIs. Even if the polymer structure contains a hinge oxygen atom, the polymer still has a large specific surface area and offers high gas permeability. The PIM-PI KAUST-PI-1 synthesized from 1,4-diamino-2,3,5,6-tetramethylbenzene as diamine exhibits excellent separation performance for many gas pairs above the upper bound in the 2008 Robeson plot.

Taking into account the aforesaid, combining different structural motifs allows one to synthesize new polymers with improved sorption and gas transport properties. In most cases the carbocyclic units incorporated into the polymer backbone have a synergistic effect with other structural blocks. Other methods for combining the advantages of different classes of polymers in order to obtain materials with specified properties include copolymerization of monomers of different natures and blending of polymers.

## 9. Conclusions and Future Outlook

Diverse synthetic routes to carbocyclic and heterocyclic structures made it possible to synthesize a variety of polymers with cyclic moieties in the backbone and in side chains. Although such polymers have long been studied, researchers continue to develop methods for incorporating cyclic units. In particular, microporous glassy TB polymers and CANAL polymers were for the first time synthesized quite recently. Only two examples of PNBs bearing 9,10-dihydroanthracene moieties have been reported until very recently, and these polymers were not considered as microporous materials.

An analysis of the sorption and gas transport properties of the polymers in question suggests that incorporation of carbocyclic moieties into the backbone and side chains of glassy polymers is a powerful tool for simultaneously increasing the free volume, specific surface area, and gas permeability. This effect can be controlled by varying the size and structural rigidity of the cyclic units. Larger carbocycles not unexpectedly cause a greater increase in microporosity compared to smaller carbocycles. Considering side chain modification, the strongest effects were observed for indane-based substituents (for PAs) and rigid polycyclic structures (for PNBs). Polymers from various classes illustrate the effect of the rigidity of the bond between the carbocycle and the backbone on the properties of the polymers; namely, the less rotatable the bond, the more pronounced the effect. It is observed both for carbocycles in side chains and in backbones. So, among PNBs, the carbocycle-substituted polymers with the largest microporosity and highest gas permeability are those with non-rotable substituents (e.g., 9,10-dihydroanthracene moieties). Among PIs, PIMs, and TB polymers with incorporated carbocyclic moieties in the main chain, the best characteristics are achieved for the polymers containing rigid, non-rotable polycyclic structures (e.g., triptycene, ethanoanthracene or pentiptycene motifs, or spiro polycycles). The chemical nature of carbocycles influences both gas permeability and specific surface area. To increase these parameters, it is appropriate to incorporate rigid, bulky polycyclic aliphatic moieties. Nevertheless, in spite of the possibility for inter-chain π–π stacking interaction to occur, the presence of side-chain aromatic carbocycles rigidly bound to the polymer backbone does not preclude an increase in microporosity. For instance, PNBs bearing 9,10-dihydroanthracene side groups exhibit excellent sorption properties and record-high gas transport characteristics. In some cases, structure modification with carbocycles is more efficient than with trimethylsilyl groups from the standpoint of increasing specific surface area and gas permeability. The gas permeability of certain PAs bearing carbocyclic substituents is higher than that of poly(1-trimethylsilylpropyne-1). It is expected that future progress in the synthetic methods for introducing carbocyclic moieties into the polymer structure will help design microporous materials.

**Figure 6:**
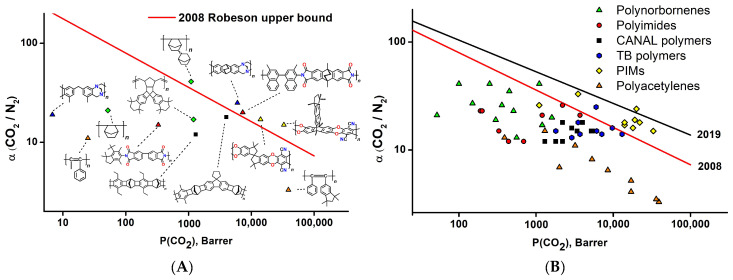
Robeson’s plot for CO_2_/N_2_ gas pair. (**A**): effect of incorporation of carbocycles on gas transport characteristics, (**B**): positions of different classes of polymers bearing carbocyclic moieties in the Robeson plot.

Not only gas permeability, but, in some cases, selectivity for gas separation can be improved upon incorporating carbocyclic moieties into the polymer structure. The effect for the CO_2_/N_2_ pair can be exemplified by the CANAL polymers, TB polymers, and PNBs (Figure 6A). By and large, polymers with carbocyclic substituents combine high gas permeability and selectivity for separation of this gas pair (Figure 6B). Moreover, carbocyclic substituents have a synergistic effect with other methods for increasing the microporosity and gas permeability. For instance, structure modification by simultaneously introducing carbocyclic substituents and CF_3_ groups leads to an increase in specific surface area and improves gas permeability and selectivity for separation of CO_2_-containing gas pairs. Polymers bearing oxygen- and nitrogen-containing heterocycles are capable of a specific dipole–quadrupole interaction between the CO_2_ molecule and the polymer matrix. Many such polymers exhibit high selectivities for separation of CO_2_/CH_4_ and CO_2_/N_2_ mixtures, and in some cases their gas separation parameters are above the upper bound in Robeson’s plot (Figure 6A). The synergistic effect also manifests itself when combining different-type carbocyclic moieties in the polymer structure. It is believed that future research activity will be aimed at extending studies of synergistic effects and attempting to incorporate, e.g., trimethylsilyl groups into the structure of PNBs bearing carbocycles rigidly bound to the backbone. Probably, such polymers will be characterized by even larger specific surface area and higher gas permeability.

Relaxation aging remains a severe drawback of polymers bearing carbocyclic moieties and other microporous glassy polymers, although ladder polymers with polycyclic frameworks incorporated into the backbone demonstrate a kind of aging resistance. Other methods to reduce the aging effect include, e.g., cross-linking of polymers and filling of polymer matrices [113,114]. One can expect that they will be useful in the design of aging-resistant, highly permeable glassy polymers based on new polymeric systems with carbocyclic moieties.

## Figures and Tables

**Table 2:** Gas transport properties of indane-containing polyacetylenes ^a^.

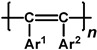		*P*, Barrer				α_i/j_	
Ar^1^	Ar^2^	N_2_	O_2_	CO_2_	CH_4_	O_2_/N_2_	CO_2_/N_2_
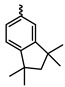	Ph	12,000	14,000	39,000	23,000	1.2	3.3
	*p*-C_6_H_4_F	16,000	18,000	47,000	33,000	1.2	3.0
	*m*-C_6_H_4_F	12,000	14,000	35,000	28,000	1.2	2.9
	*m*,*p*-C_6_H_3_F_2_	17,000	19,000	44,000	35,000	1.1	2.7
	*m*,*m*-C_6_H_3_F_2_	13,000	15,000	36,000	29,000	1.2	2.8
	*p*-C_6_H_4_Cl	9200	12,000	35,000	23,000	1.3	3.8
	*p*-C_6_H_4_Br	9200	11,000	33,000	22,000	1.2	3.6
	*p*-C_6_H_4_Me	4100	5700	17,000	10,000	1.4	4.1
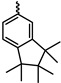	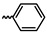	10,000	13,000	36,000	25,000	1.2	3.5
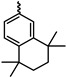	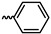	2700	3900	11,000	6600	1.4	4.1
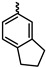	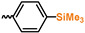	170	450	2000	470	2.7	12
	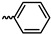	30	86	390	60	2.9	13

^a^ The data were taken from [31,39].

**Table 4:** Gas transport properties of PNBs bearing 9,10-dihydroanthracene side substituents.

Polymer ^a^	*P*, Barrer				α_i/j_		FFV, %	Ref.
	N_2_	O_2_	CO_2_	CH_4_	O_2_/N_2_	CO_2_/N_2_		
MP-NBAnt	2.4	12	99	4.2	5.0	41	19	[42,43]
MP-NBPent	72	210	1200	140	2.9	17	24	[42,43]
AP-NBAnt	43	87	560	62	2.0	13	24	[42,43]

^a^ The structures of the polymers are presented in Figure 1.

**Table 5:** Gas transport properties of polymers based on nadimides.

Polymer	*P*, Barrer				α_i/j_		FFV, %	Ref.
	N_2_	O_2_	CO_2_	CH_4_	O_2_/N_2_	CO_2_/N_2_		
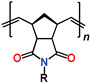								
R								
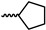	0.45	1.9	8.8	0.54	4.2	20	15	[51]
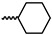	0.43	2.3	9.2	0.48	5.3	21	15	[51]
	0.6	2.1	18	1.1	3.5	30	–	[52]
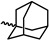	0.5	1.6	8.4	0.58	3.2	17	–	[52]
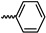	0.3	1.4	11	0.54	4.7	37	19	[53]
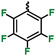	1.6	6.1	25	1.4	3.8	16	20	[53]
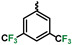	4.2	14	67	4.3	3.3	16	20	[53]
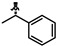	0.18	0.98	6.0	0.22	5.4	33	–	[54]
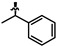	0.14	1.1	6.2	0.21	7.9	44	–	[54]
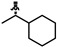	0.26	1.3	12	0.36	5.0	46	–	[54]
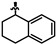	0.22	1.3	7.4	0.31	5.9	34	–	[54]
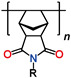								
R								
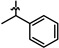	0.83	3.7	37	1.3	4.5	45	8	[54]
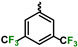	43	120	1400	47	2.8	33	14	[54]

**Table 6:** Gas transport properties and BET surface areas of PNBs with side chain porosity.

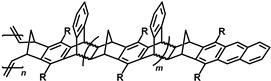									
R	*m*	*P*, Barrer				α_i/j_		*S*_BET_, m^2^ g^−1^	Ref.
		N_2_	O_2_	CO_2_	CH_4_	O_2_/N_2_	CO_2_/N_2_		
CF_3_	–	–	–	–	–	–	–	780	[57]
OMe	–	–	–	–	–	–	–	590	[57]
CF_3_	–	2000	4000	19,000	3100	2.0	9.3	700	[58]
OMe	–	150	410	2900	270	2.7	19	150	[58]
OMe	0	3.6	13	83	5	3.6	23	30	[59]
OMe	1	5	26	180	9	5.2	36	150	[59]
OMe	2	36	110	830	58	3.1	23	430	[59]
OMe	3	110	310	2300	190	2.8	21	570	[59]
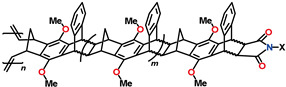						**X**		***S*_BET_, m^2^ g^−1^**	**Ref.**
						Ph		330	[57]
						CH_2_CH_2_OH		550	[57]

**Table 7:** Gas transport properties and BET surface areas of PIs.

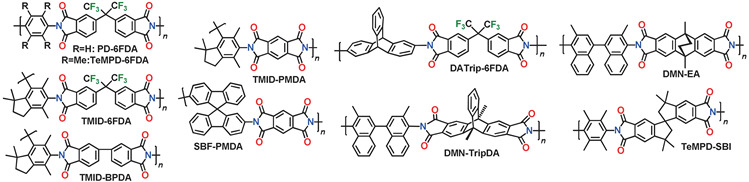									
Polymer	*P*, Barrer				α_i/j_		*S*_BET_, m^2^ g^−1^	FFV, %	Ref.
	N_2_	O_2_	CO_2_	CH_4_	O_2_/N_2_	CO_2_/N_2_			
PD-6FDA	0.8	4.2	15	0.29	5.3	19	–	16	[60]
TeMPD-6FDA	36	120	440	28	3.4	12	–	18	[60]
TMID–BPDA	22	83	330	24	3.8	15	–	17	[61]
TMID–6FDA	59	200	690	51	3.4	12	–	17	[61]
TMID-PMDA	58	230	1200	76	4.0	21	–	21	[62]
DATrip-6FDA	8.1	39	190	6.2	4.8	23	68	23	[63]
SBF-PMDA	8.5	36	200	9.1	4.2	23	320	–	[64]
TeMPD-SBI	84	270	2200	170	3.2	26	690	–	[65]
DMN-TripDA	180	780	3700	220	4.3	21	760	–	[66]
DMN-EA	370	1400	7300	460	3.7	20	620	–	[67]

**Table 8:** Gas transport properties and BET surface areas of PIMs.

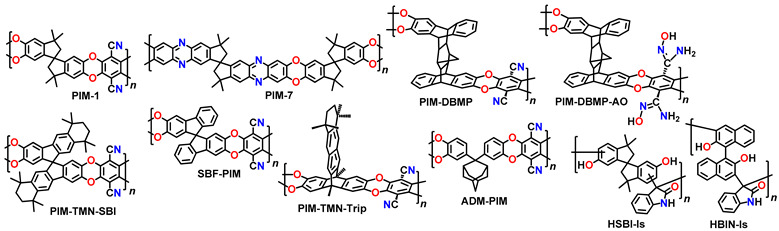								
Polymer	*P*, Barrer ^a^				α_i/j_		*S*_BET_, m^2^ g^−1^	Ref.
	N_2_	O_2_	CO_2_	CH_4_	O_2_/N_2_	CO_2_/N_2_		
PIM-1	820	2300	14,000	1400	2.8	17	720	[80]
PIM-7	42	190	1100	62	4.5	26	680	[87]
SBF-PIM	790	2600	14,000	1100	3.4	18	800	[88]
PIM-TMN-SBI	1100	3200	18,000	2100	3.0	16	1000	[81]
PIM-TMN-Trip	2200	7500	33,000	3400	3.4	15	1100	[81]
PIM-1_0.9_/PIM-DBMP_0.1_ ^b^	1000	3300	19,000	1600	3.3	19	800	[82]
PIM-1_0.75_/PIM-DBMP_0.25_ ^b^	1200	3900	22,000	1900	3.3	18	760	[82]
PIM-1_0.5_/PIM-DBMP_0.5_ ^b^	1200	4100	22,000	1800	3.4	18	830	[82]
PIM-1_0.25_/PIM-DBMP_0.75_ ^b^	830	3400	20,000	1500	4.1	24	730	[82]
PIM-1_0.1_/PIM-DBMP_0.9_ ^b^	–	–	–	–	–	–	820	[82]
PIM-DBMP	–	–	–	–	–	–	790	[82]
PIM-DBMP-AO	110	690	3500	110	6.5	33	650	[82]
PIM-1_0.75_/ADM-PIM_0.25_ ^b^	120	470	2100	150	3.9	17	720	[83]
PIM-1_0. 5_/ADM-PIM_0.5_ ^b^	86	360	1800	100	4.2	21	710	[83]
PIM-1_0.25_/ADM-PIM_0.75_ ^b^	68	290	1700	82	4.3	25	710	[83]
ADM-PIM	41	230	1100	48	5.7	27	700	[83]
HSBI-Is	27	130	680	36	4.7	25	520	[89]
HBIN-Is	0.79	5.7	27	0.37	7.2	35	57	[89]

^a^ Gas transport properties of methanol-treated films. ^b^ Copolymer.

**Table 9:** Gas transport properties and BET surface areas of TB polymers.

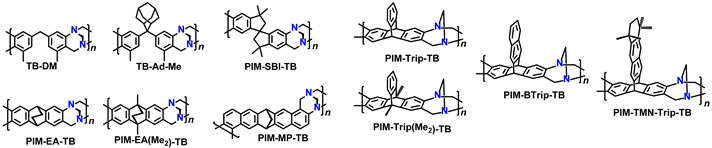								
Polymer	*P*, Barrer ^a^				α_i/j_		*S*_BET_, m^2^ g^−1^	Ref.
	N_2_	O_2_	CO_2_	CH_4_	O_2_/N_2_	CO_2_/N_2_		
TB-DM	0.38	–	6.8	0.24	–	19	38	[8]
TB-Ad-Me	120	440	1800	160	3.6	15	620	[92]
PIM-SBI-TB	230	720	2900	450	3.1	13	750	[96]
PIM-EA(Me_2_)-TB	530	2200	7100	700	4.2	14	1000	[96]
PIM-EA-TB	240	–	6000	370	–	25	850	[97]
PIM-MP-TB	200	1000	3500	260	5.0	18	740	[91]
PIM-Trip-TB	630	2700	9700	910	4.3	16	900	[98]
PIM-Trip(Me_2_)-TB	260	1000	3700	350	3.9	14	930	[93]
PIM-BTrip-TB	930	3300	13,000	1400	3.6	14	870	[94]
PIM-TMN-Trip-TB	400	2000	6100	710	5.1	15	–	[81]

^a^ Gas transport properties of methanol-treated films.

**Table 10:** Properties of CANAL polymers.

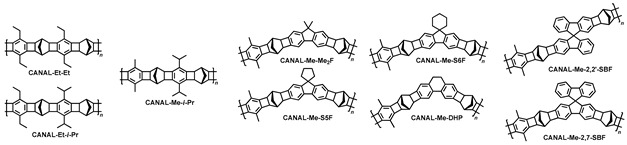									
Polymer	*P*, Barrer				α_i/j_		*S*_BET_, m^2^ g^−1^	FFV, %	Ref.
	N_2_	O_2_	CO_2_	CH_4_	O_2_/N_2_	CO_2_/N_2_			
CANAL-Et-Et	110	310	1300	270	2.8	12	–	23	[101]
CANAL-Et-*i*-Pr	150	410	1800	350	2.7	12	–	26	[101]
CANAL-Me-*i*-Pr	190	490	2200	380	2.5	12	–	27	[101]
CANAL-Me-Me_2_F	370	1400	5400	570	3.8	15	1200	–	[104]
CANAL-Me-S5F	220	970	4000	320	4.4	18	1100	–	[104]
CANAL-Me-S6F	190	730	2900	290	3.9	16	940	–	[104]
CANAL-Me-DHP	230	790	3400	400	3.4	15	870	–	[104]
CANAL-Me-2,7-SBF	350	1100	5200	660	3.2	15	690	–	[105]
CANAL-Me-2,2′-SBF	130	450	2200	220	3.5	18	510	–	[105]

**Table 11:** Properties of polymers containing CANAL units.

Polymer	*P*, Barrer				α_i/j_		*S*_BET_, m^2^ g^−1^	FFV, %	Ref.
	N_2_	O_2_	CO_2_	CH_4_	O_2_/N_2_	CO_2_/N_2_			
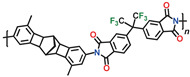	7	33	160	4.8	4.7	23	200	25	[108]
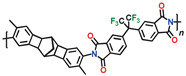	21	81	420	16	3.9	20	220	26	[108]
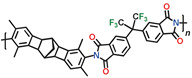	91	320	1700	110	3.5	19	530	29	[108]
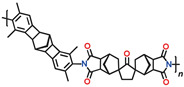	95	330	1100	120	3.5	12	570	25	[109]
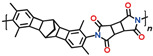	56	170	930	60	3.0	17	530	17	[110]
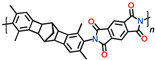	79	280	1400	91	3.5	18	620	18	[110]
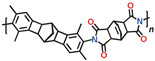	110	390	2000	130	3.6	18	610	19	[110]
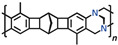	97	460	1700	120	4.8	18	880	–	[111]
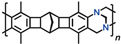	160	750	2500	210	4.6	16	990	–	[111]

**Table 12:** Properties of triptycene-containing PIM-polyimides ^a^.

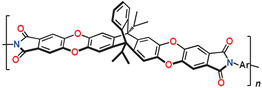							
Ar	*P*, Barrer				α_i/j_		*S*_BET_, m^2^ g^−1^
	N_2_	O_2_	CO_2_	CH_4_	O_2_/N_2_	CO_2_/N_2_	
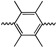	110	630	2400	110	5.9	22	750
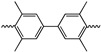	98	490	2100	100	5.0	21	740
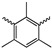	43	240	920	43	5.5	21	760
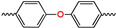	11	48	290	11	4.5	27	420
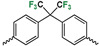	87	360	1600	77	4.1	18	650
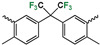	14	65	320	11	4.5	22	500
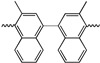	230	840	4400	350	3.7	20	840

^a^ The data were taken from [112].

## Data Availability

No new data were created or analyzed in this study. Data sharing is not applicable to this article.

## Abbreviations

The following abbreviations are used in this manuscript:

APNB	Vinyl-addition polynorbornene
BET	Brunauer–Emmett–Teller
CANAL	Catalytic arene-norbornene annulation
FFV	Fractional free volume
MPNB	Metathesis polynorbornene
PA	Polyacetylene
PI	Polyimide
PIM	Polymer of intrinsic microporosity
PNB	Polynorbornene
PTMSP	Poly(1-trimethylsilyl-1-propyne)
TB	Tröger’s base
TR	Thermally rearranged
